# Prognostic impact of inducible ischaemia in ischaemic left ventricular dysfunction: the REVIVED-BCIS2 trial

**DOI:** 10.1093/eurheartj/ehae844

**Published:** 2024-12-11

**Authors:** Holly Morgan, Muhummad Sohaib Nazir, Matthew E Li Kam Wa, Gerry P McCann, John P Greenwood, Adam K McDiarmid, Matthew Dodd, Matthew Ryan, Divaka Perera, Amedeo Chiribiri

**Affiliations:** British Heart Foundation Centre of Research Excellence at the School of Cardiovascular and Metabolic Medicine & Sciences, King’s College London, Westminster Bridge Road, London SE1 7EH, UK; School of Biomedical Engineering & Imaging Sciences, King’s College London, Westminster Bridge Road, London SE1 7EH, UK; Royal Brompton Hospital, Sydney St, London SW3 6NP, UK; British Heart Foundation Centre of Research Excellence at the School of Cardiovascular and Metabolic Medicine & Sciences, King’s College London, Westminster Bridge Road, London SE1 7EH, UK; University of Leicester and the NIHR Leicester Biomedical Research Centre, University Rd, Leicester LE1 7RH, UK; Leeds Teaching Hospitals NHS Trust, UK and Baker Heart and Diabetes Institute, Melbourne, Australia; Newcastle Hospitals NHS Foundation Trust, Newcastle upon Tyne NE7 7DN, UK; London School of Hygiene and Tropical Medicine, Keppel St, London WC1E 7HT, UK; British Heart Foundation Centre of Research Excellence at the School of Cardiovascular and Metabolic Medicine & Sciences, King’s College London, Westminster Bridge Road, London SE1 7EH, UK; Guy’s and St Thomas’ NHS Foundation Trust, Westminster Bridge Road, London SE1 7EH, UK; British Heart Foundation Centre of Research Excellence at the School of Cardiovascular and Metabolic Medicine & Sciences, King’s College London, Westminster Bridge Road, London SE1 7EH, UK; Guy’s and St Thomas’ NHS Foundation Trust, Westminster Bridge Road, London SE1 7EH, UK; School of Biomedical Engineering & Imaging Sciences, King’s College London, Westminster Bridge Road, London SE1 7EH, UK

**Keywords:** Ischaemia, Ischaemic heart failure, Percutaneous coronary intervention, Magnetic resonance imaging

## Introduction

Retrospective observational data suggest that the presence and extent of inducible myocardial ischaemia predict future adverse cardiac events in patients with ischaemic heart disease. However, several randomized trials have failed to identify an association between the burden of inducible ischaemia and outcomes in patients with stable coronary artery disease (CAD) assigned to medical therapy alone or revascularization.^1,2^ Despite this, the presence and extent of ischaemia are commonly used to guide revascularization of stable patients. Notably, many of these analyses excluded patients with impaired left ventricular (LV) function.

REVIVED-BCIS2 was a prospective, multi-centre, randomized controlled trial, investigating whether percutaneous coronary intervention (PCI) improved clinical outcomes in patients with severe ischaemic LV dysfunction (ILVD), compared with optimal medical therapy (OMT) alone.^3,4^ Whilst ischaemia testing was not mandated by protocol, it was performed as part of routine clinical practice in selected patients. Based on these data, we sought to determine whether the presence and extent of ischaemia detected on stress perfusion cardiac magnetic resonance (CMR) imaging predict clinical outcomes and response to PCI, in patients with ILVD.

## Methods

**Figure 1:**
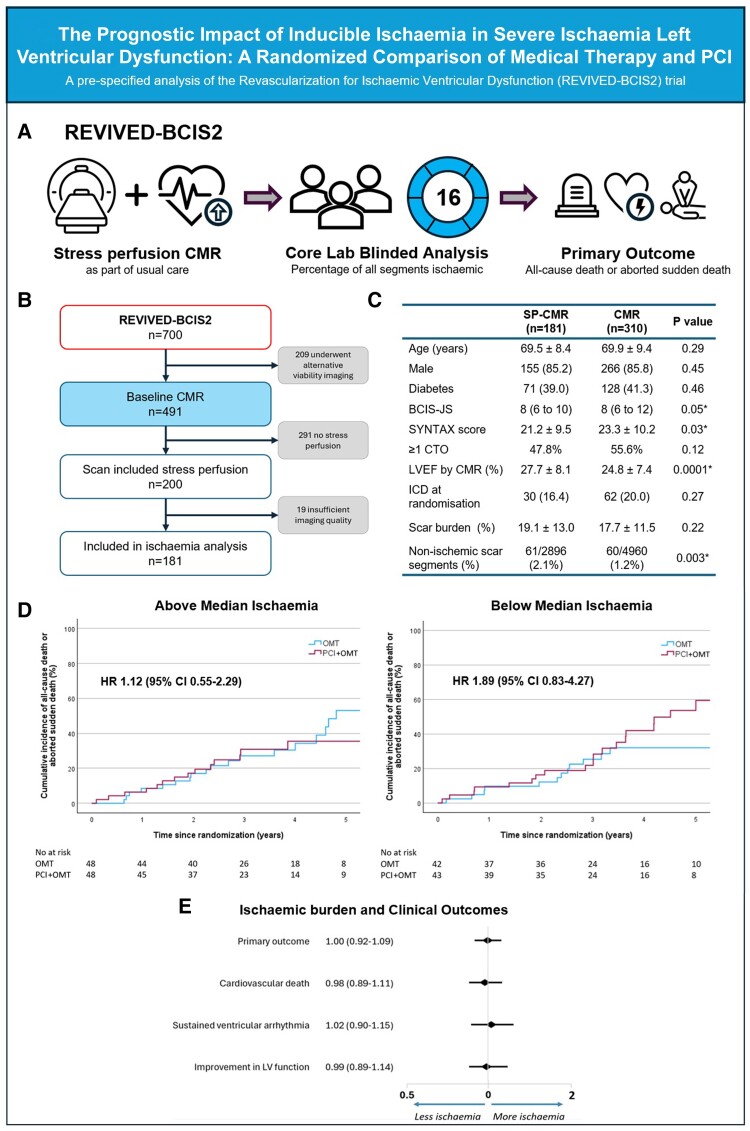
Impact of inducible ischaemia in ischaemic left ventricular dysfunction in REVIVED-BCIS2. (*A*) Methodology. (*B*) CONSORT flow chart of this analysis. (*C*) Baseline demographics of those who had undergone a stress perfusion cardiac MRI (SP-CMR) as part of usual care prior to randomization, vs. who had a CMR without (CMR). NB. No differences were identified in baseline demographics between those randomized to PCI or OMT. (*D*) All-cause death or aborted sudden death in participants assigned to PCI with OMT vs. OMT alone. Left: above median ischaemia group, including values which fall on the median (≥31%). Right: below median ischaemia group (<31%). Individual component were as follows: OMT arm—23 deaths, 13 appropriate therapies, 2 RCA; PCI arm—29 deaths, 9 appropriate therapies, 2 RCA. (*E*) Association between ischaemic burden and clinical outcomes. Unadjusted hazard ratios per 10% ischaemic burden are shown (odds ratio shown for improvement in LV function). BCIS-JS, British Cardiovascular Intervention Society jeopardy score; CTO, chronic total occlusion; ICD, implantable cardioverter defibrillator; OMT, optimal medical therapy; PCI, percutaneous coronary intervention; SYNTAX, synergy between percutaneous coronary intervention with taxus and cardiac surgery; scar burden expressed as a percentage of LV mass. Non-ischaemic scar segments = segments containing scar that was not in an ischaemic distribution

Patients enrolled in REVIVED-BCIS2 were eligible for inclusion in this analysis if they had undergone stress perfusion CMR prior to randomization. Visual segmental assessment of myocardial perfusion and late gadolinium enhancement transmurality was completed at an independent core laboratory blinded to trial-group assignment and clinical data. Segmental ischaemia was classified in a binary manner and the patient-level ischaemic burden expressed in relation to total LV mass, defined as [number of ischaemic segments]/16 ∗ 100 (*Figure 1A*).

The primary composite outcome was all-cause death or aborted sudden death [defined as an appropriate implantable cardioverter defibrillator (ICD) therapy or a resuscitated cardiac arrest (RCA)]. Secondary outcomes included cardiovascular death, heart failure (HF) hospitalizations, appropriate ICD therapies,^5^ sustained ventricular arrhythmias,^5^ and LV ejection fraction (EF) change at 6 months. Clinical outcome data were collected from the main trial and arrhythmia-specific case report forms. Death and HF hospitalization events were adjudicated by an independent clinical events committee.^3^

The statistical analysis plan was finalized prior to unblinding of the CMR core laboratory data. A time-to-event analysis was performed on the primary endpoint, with the time to the first event (or censoring) measured from randomization. The interaction between randomized assignment, ischaemic burden (treated as a linear effect) and major outcomes was assessed using a Cox proportional hazards model, adjusted for age, sex, presence of diabetes, cardiac device at baseline, extent of CAD, chronic renal failure, and baseline LVEF. Categorical demographic data are presented as counts (percentages) and continuous data as mean ± standard deviation or median [interquartile range].

## Results

In total, 200 of 700 REVIVED-BCIS2 patients underwent stress perfusion CMR, of which 181 were of sufficient diagnostic quality to be included in this analysis (*Figure 1B*). Baseline demographics are shown in *Figure 1C*. Compared to the 310 patients who underwent CMR imaging without stress perfusion (and hence were excluded from this analysis), those included had a lower SYNTAX score (21 ± 10 vs. 23 ± 10, *P* = .03) and higher LVEF (28 ± 8% vs. 25 ± 7%, *P* < .001). Ninety-one were randomized to PCI with OMT and 90 to OMT alone, with no difference in the rates of medical therapy or heart failure devices. On CMR, scar burden was 18% [9–26] and ischaemic burden was 31% [12–56]. There was no interaction between baseline Canadian Cardiovascular Society angina score and ischaemic burden (F(3–177) = 1.54, *P* = .21).

At a median of 42 months [29–61], a primary outcome event had occurred in 36 of 91 participants in the PCI arm, and 31 of 90 in the OMT arm (40% vs. 34%, HR 1.20, 95% CI .72–1.98, *P* = .49). There was no relationship between ischaemic burden, outcomes, and randomized treatment allocation (*Figure 1D* and *E*).

Across the whole cohort, there was no association between ischaemic burden and the primary outcome (HR 1.00 per 10% increase in ischaemic burden, CI .92–1.09, *P* = .94), or with cardiovascular death (HR .98 per 10%, CI .89–1.11), HF hospitalizations (HR .93 per 10%, CI .81–1.08), sustained ventricular arrhythmias (HR 1.02 per 10%, CI .90–1.15), or improvement in LV function (OR .99 per 10%, CI .89–1.14).

## Discussion

In this analysis, we have identified that in patients with severely impaired systolic function and an ischaemic aetiology, ischaemic burden was not associated with clinical outcomes or treatment assignment.

The majority of previous work on ischaemic burden and outcomes has been in all-comer populations with preserved LV function.^6,7^ Hachamovitch *et al*. identified an interaction between ischaemic burden, early revascularization, and mortality. However, this analysis was retrospective, and the effect was not seen in patients with prior myocardial infarction.^8^ Importantly, our study comprises a cohort with a higher ischaemic burden than ISCHEMIA, the Surgical Treatment for Ischemic Heart Failure (STICH) trial (both of which failed to find an association between ischaemia and outcomes), or the aforementioned large observational cohort.^6,9^ The extensive ischaemia and scar burden shown in this analysis explain the high event rate observed within the trial and also verify the ischaemic aetiology of the patients enrolled into REVIVED-BCIS2.

In ischaemic left ventricular dysfunction, the ventricle has likely been subjected to chronic ischaemia leading to hibernation, as well as previous infarction.^10^ The relevance of stress hypoperfusion in a ventricle that has adapted to ischaemia is therefore unclear. It may be that in this advanced disease state, stress hypoperfusion (as opposed to true physiological ‘ischaemia’) is unable to further stratify risk nor identify those who might benefit from revascularization.

Limitations include that ischaemia testing was discretionary within REVIVED-BCIS2 and therefore this analysis only contains 26% of the trial population. As a *post hoc* analysis, the study was not prospectively powered, although the hazard ratio of 1.00 suggests that the risk of a type II error would be low. Finally, follow-up ischaemia testing was not undertaken, and therefore we cannot assess how the baseline ischaemic burden was altered by revascularization, or the association between residual ischaemia burden and subsequent outcomes.

## Conclusions

The REVIVED-BCIS2 cohort is the largest to date of stress perfusion CMR in severe ILVD, randomized to PCI and OMT, vs. medical therapy alone. In this cohort, ischaemic burden was not associated with clinical outcomes. Furthermore, no association was seen between ischaemic burden, clinical outcomes, and randomized treatment assignment.

## Declarations

### Data Availability

The data that support the findings of this study are available from the corresponding author upon reasonable request.

### Pre-registered Clinical Trial Number

The pre-registered clinical trial number is NCT01920048.
